# Embryogenesis of European Radish (*Raphanus sativus* L. subsp. *sativus* Convar. Radicula) in Culture of Isolated Microspores In Vitro

**DOI:** 10.3390/plants10102117

**Published:** 2021-10-06

**Authors:** Elena Victorovna Kozar, Elena Alekseevna Domblides, Alexsey Vasilevich Soldatenko

**Affiliations:** Federal State Budgetary Scientific Institution Federal Scientific Vegetable Center (FSBSI FSVC), VNIISSOK, 143072 Moscow Region, Russia; edomblides@mail.ru (E.A.D.); alex-soldat@mail.ru (A.V.S.)

**Keywords:** radish, microspores culture, embryogenesis, isolated microspores

## Abstract

The European radish is one of the most unresponsive crops in the *Brassicaceae* family to embryogenesis in *in vitro* microspore culture. The aim of this work was to study the process of embryogenesis of European radish and its biological features. In this study, the embryogenesis of European radish is described in detail with illustrative data for the first time. For the first time for the entire family *Brassicaceae*, the following were found: microspores with intact exines with ordered-like divisions; microspores completely free of exines; and a new scheme of suspensors attachment to the apical parts of embryoids. The morphology of double and triple twin embryoids was described, and new patterns of their attachment to each other were discovered. Uneven maturation of European radish embryoids at all stages of embryogenesis was noted. The period of embryoid maturation to the globular stage of development corresponded, in terms of time, to the culture of *B. napus*, and into the cotyledonary stage of development, maturation was faster and amounted to 17–23 days. The rate of embryoid development with and without suspensors was the same.

## 1. Introduction

Isolated microspore culture *in vitro* (IMC) is used to produce doubled haploids (DH-plants), which are necessary to speed up the breeding process and are very convenient for genetic research [1]. This technology began to develop a long time ago, but its potential continues to be unlocked, both for practical applications and gaining fundamental knowledge. For example, the development of some microspores is very similar to zygotic embryogenesis, and relatively recently, protocols have been developed in which a significant proportion of microspores are able to develop according to the zygotic type, making it possible to study embryogenesis carefully from the earliest stages of development [2,3,4,5,6,7,8,9]. Embryogenesis in microspore culture has been studied very extensively for the well-responsive crops to the IMC technology. Indeed, many statistical data are needed to study the biological mechanisms in order to draw reliable conclusions. *In vitro* microspore culture of one of the most responsive cultures to embryogenesis, *B. napus*, has been used to study totipotency [4,10,11], hormone signaling [12,13,14,15,16,17], cell walls [18,19,20,21], and the role of the suspensor in embryoid formation [5,15]. Nevertheless, there are still many unknowns in this field of knowledge, scientists’ opinions do not coincide in all aspects, and new evidence and refutations of hypotheses continue to emerge. In addition, any additional knowledge about this process in new crops can significantly increase knowledge and advance research.

The European radish is a difficult crop in terms of obtaining DH-plants using IMC technology because the responsiveness of its microspores is extremely poor. The species *R. sativus* L. is divided into three subspecies: *sativus* L. (European radish), sinensis Sazon.et Stankev. (Chinese radish), and acanthiformis (Blanch.) Stankev. (Japanese radish). In the literature, obtaining doubled haploids of the species *R. sativus* L. has been reported sporadically, with most articles focusing on subsp. acanthiformis (Blanch) Stankev., while information about subsp. *sativus* L. was covered in only one article [22,23,24,25]. The first article about subsp. acanthiformis (Blanch) Stankev. was published back in 1996, describing the successful production of doubled haploids in *in vitro* microspore culture, with an embryoid yield of 8.3 ± 2.3 per Petri dish [22]. Further, obtaining doubled haploids of this subspecies was described for several other cultivars, but a higher embryoid yield could not be obtained. For subsp. *sativus* L., the first attempts to obtain doubled haploids in *in vitro* microspore culture were described in 2017, but the studies only produced callus in a liquid medium, i.e., embryoids were not formed [25]. In our laboratory, we developed an IMC protocol for obtaining doubled haploids of the European radish with which we were first able to complete a full cycle of obtaining DH-plants of European radish (subsp. *sativus* L.). As a result, we have a unique opportunity to add knowledge to the embryogenesis process for one of the most nonresponsive cultures to IMC technology in the *Brassicaceae* family. Moreover, morphological features of the embryogenesis of *R. sativus* L. species in *in vitro* microspore culture have not been previously described for any of the subspecies.

The aim of the study was to investigate the pathways of the main stages and biological features of European radish embryogenesis in isolated microspore culture *in vitro* from the first day to the cotyledon stage of embryoid development, when ready for transfer to a solid medium for regeneration.

## 2. Results

The European radish is one of the most unresponsive crops in the Brassicaceae family for embryogenesis. Currently, the most successful protocol for obtaining doubled haploids in radish microspore culture is described in Protocol [26]. The protocol is still not highly efficient and can produce no more than eight embryoids per petri dish [27]. For this reason, each embryoid is of great value, and in order to not disturb their development, we observed the formation of embryoids only in live culture. Observations in live liquid culture cannot provide much information, but we used just this because the since the efficiency of the technique is low, we could not risk wasting any of the obtained embryogenic structures on fixed preparations or observe the individual development of each structure, since this requires introducing elements into the technique that reduce the embryoid yield. In the European radish, the responsive stages for gametic embryogenesis are late uninucleate vacuolized microspores and early binucleate pollen. For inoculation, we selected buds with the maximum number of such stages and inoculation was performed according to protocol [20].

**Figure 1:**
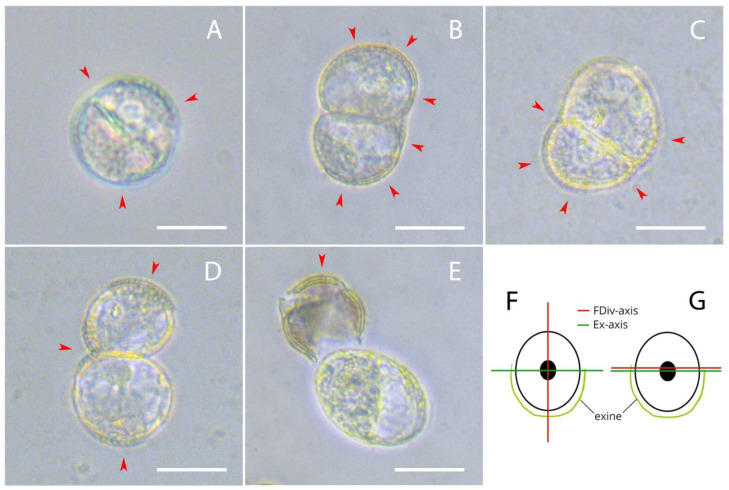
Division in a culture of European radish microspores on the second day of culture: (**A**) Microspore with intact exine with classical equal division; (**B**,**F**) microspore with a single exine rupture and a cell division plane perpendicular to the exine rupture with limited loss of adhesion; (**C**,**G**) microspore a plane of unequal cell division with parallel to exine rupture without loss of adhesion; (**D**) microspores with ruptured exine in three places with extensive loss of adhesion between cells; (**E**) microspore without exine. The red arrows indicate the exine. The axis of the plane of exine rupture is indicated as Ex-axis and the axis of the plane of first cell division is FDiv-axis. Bars = 20 µm.

From the second day after inoculation, we observed the first microspore divisions. In the culture, we could observe microspores with an intact exine with symmetric division (Figure 1A) [21,22,23,24,25]; microspores with a single ruptured exine and a cell division plane perpendicular to the exine rupture with limited loss of adhesion (Figure 1B,F), from which twin embryoids can form [27]; microspores with a single ruptured exine where the axis of the ruptured exine was parallel to the axis of the first unequal cell division without loss of adhesion (Figure 1C,G), which likely formed further embryoids with suspensors [27]; microspores with a ruptured exine in three places with extensive loss of adhesion between cells (Figure 1D) and microspores that were completely free of an exine (Figure 1E).

**Figure 2:**
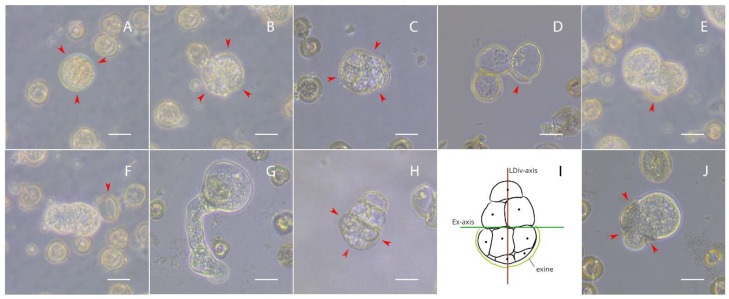
Divisions in European radish microspore culture on days 3 to 5 of culture: (**A**–**C**) Divisions of microspores with intact exine; (**D**) highly vacuolated cell divisions of microspore; (**E**) division with dense cells; (**F**) “dense” cell division of microspores without exine; (**G**) vacuolized structures of irregular shape; (**H**,**I**) microspore with less vacuolated and small cells in the area of contact with exine and with vacuolated cells in the area free of exine; (**J**) exine remnants pulled over the embryogenic structures, contributing to their aberrant shape. The red arrows indicate the exine. The axis of the plane of exine rupture is indicated as Ex-axis and the axis of the long plane of division is LDiv-axis. Bars = 20 µm.

**Figure 3:**
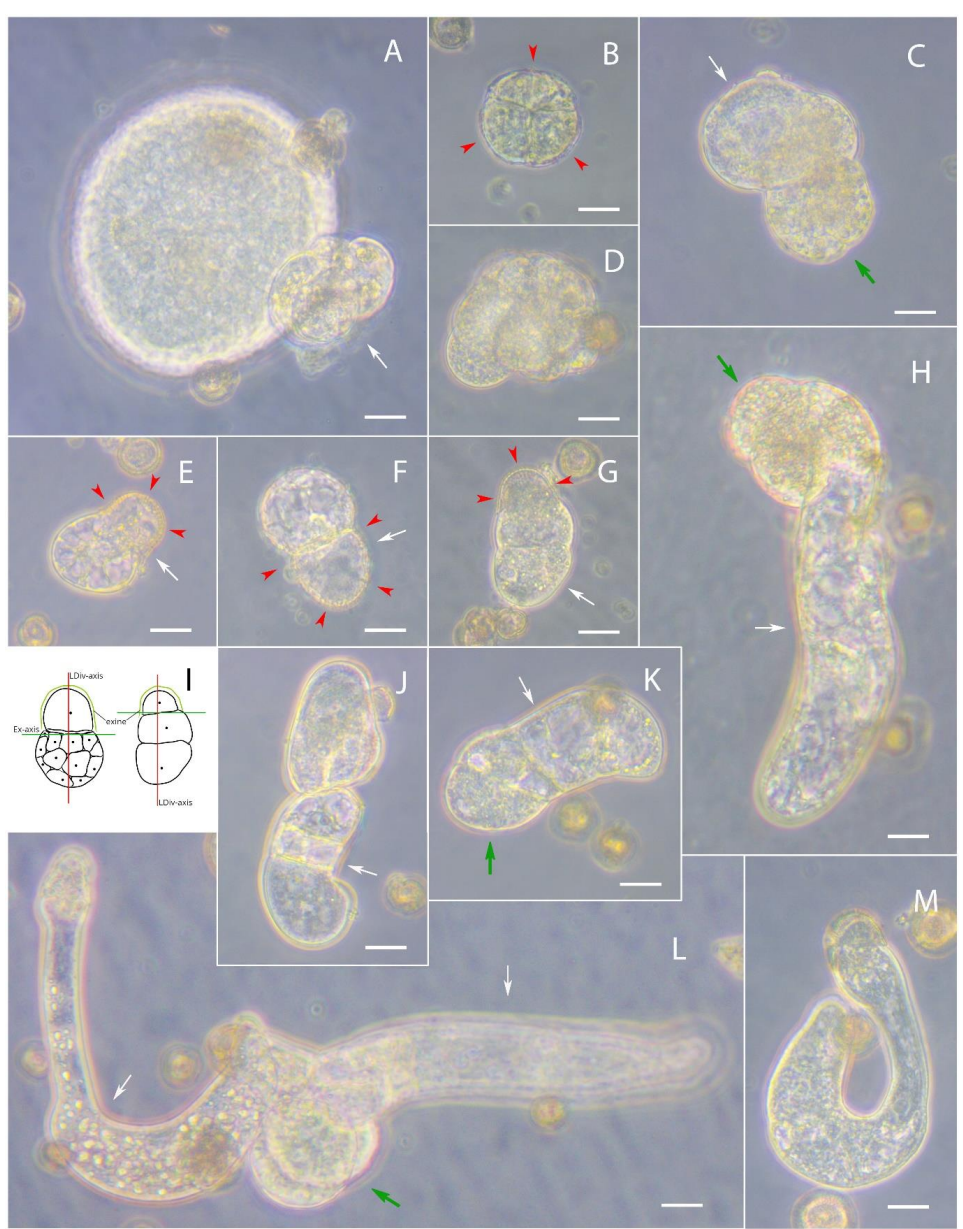
Divisions in a culture of European radish microspores on day 7 of culture: (**A**) Globular-shaped embryoid with aberrant suspensor; (**B**) structure with few cell divisions and unbroken exine; (**C**) array of dense cells and cells with increased vacuolization not properly shaped; (**D**) array of dense cells not properly shaped; (**E**,**F**) divisions with a large vacuolated cell in the exine contact area and a denser group of cells in the exine-free part; (**G**) suspensor cells; (**H**) structure with the embryoid proper and suspensor; (**I**) schematic representation of different types of cell division; (**J**) budding twin embryo; (**K**) suspensor cells with first divisions of the embryoid proper; (**L**) suspensor structure with budding twin embryo; (**M**) suspensor. The red arrows indicate the exine. The green arrows are the embryoid and the white arrows are the suspensor. The axis of the plane of exine rupture is specified as Ex-axis and the axis of the long plane of division is LDiv-axis. Bars = 20 µm.

We then observed great variety in the types of different divisions in culture, which differed in the shape and density of cell structures. It was noticeable that the development of divided microspores was not uniform, and similar divisions of microspores could be observed on both days 4 and 7 of culture (Figure 2A–C,H and Figure 3B,E,F). On days 3–5 of culture, the most compact was the structures with an intact exine (Figure 2A–C). Interestingly, in microspores with an intact exine, we also observed divisions that were similarly ordered as in the formation of embryoids with suspensors, but we were unable to further trace the fate of such cell structures (Figure 2C). Microspores with extensive loss of cell adhesion showed different division patterns. So, in one case, the cells were highly vacuolated and did not look embryogenic (Figure 2D), and in another case, the cells looked quite dense and had a chance of further development into embryoids (Figure 2E). Microspores that were completely free of an exine were able to divide into ‘dense’ cells (Figure 2F) and vacuolized structures of irregular shape (Figure 2G). Part of the microspores formed less vacuolated and small cells in the area of contact with the exine, while the exine-free part of the cells was more similar to those that could later form the suspensor, with the long plane of divisions (polarity/future apical–basal axis) being perpendicular to the break of the exine plane (Figure 2H,I). Similarly, divisions were found where exine remnants pulled over the embryogenic structures, contributing to their aberrant shape (Figure 2J).

After a week of culture, microspores with few cell divisions were found in culture, just as on days 3–5 of culture (Figure 3B), indicating that the initiation of divisions was delayed or the embryogenic structure had died. Globular-shaped embryoids both with and without various rudimentary outgrowths similar to an aberrant suspensor were also present in the culture (Figure 3A).

Some structures did not have a regular shape and looked like an array of dense cells (Figure 3D), or between of dense cells array were noted cells with increased vacuolization which for further represented aberrant suspensors (Figure 3C). Some microspores formed a large vacuolated cell in the exine contact area and a denser group of cells in the exine-free part (Figure 3E,F). This was similar to the initial stage of development of an embryoid with a suspensor, where the formation of the embryoid proper and the suspensor occurred simultaneously. In Figure 3G,H,K, the development is more like zygotic development, where a number of cells of the suspensor are formed first, and only then does the embryoid proper begin to form at one end of the suspensor. The figures show that these structures are at different stages of development, starting with the initial one, where only the suspensor cells are formed (Figure 3G), then the first divisions of the embryoid proper (Figure 3K), and the structure where the embryoid proper and suspensor are already clearly visible (Figure 3H). Furthermore, the images show that the cells of the suspensor appear less dense, which may indicate that they are more vacuolated than the cells of the embryoid proper. Once again, we note that these images were taken on the same day of culture, which confirms the different rates of formation of the structures. It is also important to note that in all structures where we could identify remnants of the ruptured exine, the axis of the long division plane was always perpendicular to the rupture of the exine plane (Figure 3E–G,I). Formations of very irregular shapes were also present in the culture (Figure 3J,L,M). We assume that the structure in Figure 3M is a suspensor, Figure 3J is a budding twin embryo, and Figure 3L is a suspensor structure and budding twin embryo. Unfortunately, due to the mobility of the liquid culture of microspores, we were unable to trace the exact fate of such structures, but their presence is of interest for further investigation of the different embryogenesis pathways.

**Figure 4:**
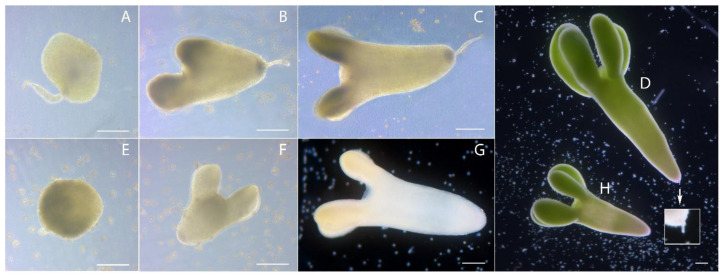
Developmental stages of European radish embryoids in microspore culture *in vitro* (classical stages of embryoids): (**A**) Globular stage of embryoid with suspensor; (**B**) heart-shaped stage of embryoid with suspensor; (**C**) torpedo-shaped stage of embryoid with suspensor; (**D**) cotyledonous stage of embryoid with suspensor; (**E**) globular stage of embryoid; (**F**) heart-shaped stage of embryoid; (**G**) torpedo-shaped stage of embryoid; (**H**) cotyledonous stage of embryoid. Bars = 200 µm.

The globular stage of embryoid development was formed by approximately 7–13 days of culture (Figure 4A,E), heart-shaped by 12–15 days of culture (Figure 4B,F), torpedo-shaped by 13–18 days of culture (Figure 4C,G), and cotyledonous by 17–23 days of culture (Figure 4D,H). Embryoids developed with (Figure 4A–D) or without a suspensor (Figure 4E–H) and we observed no difference in the developmental rate of embryoids dependent on the presence of a suspensor. 

**Figure 5:**
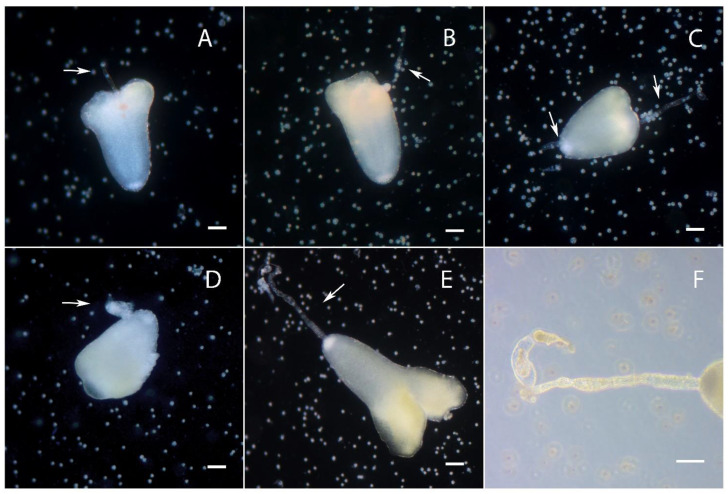
European radish embryoids obtained in microspore culture *in vitro*, with different types of suspensor attachment. White arrows indicate suspensors: (**A**) Embryoid with attachment of the suspensor to the apical part between the cotyledons; (**B**) embryoid with attachment of the suspensor to the apical part from the lateral side; (**C**) embryoid formed in the central part of the suspensor with the apical–basal axis of the embryo parallel to the axis of the suspensor, so that one suspensor is attached to the basal part of the embryoid and the other to the apical part; (**D**) embryoid with the suspensor attached to the basal part laterally; (**E**) embryoid with the suspensor attached to the central axis of the basal part of the embryoid; (**F**) suspensor with irregular cell septa, and varying cell lengths. Bars = 100 µm.

In addition to the classical development of embryoids, we observed numerous atypical elements. Of particular interest is the diversity of embryoid morphology with suspensors. We observed the attachment of the suspensor to the apical part of the embryoid between the cotyledons (Figure 5A) or to the apical part from the lateral side (Figure 5B). In embryoids that were formed in the central part of the suspensor, we first observed the orientation of the apical–basal axis of the embryoids parallel to the axis of the suspensor, so that one suspensor was attached to the basal part of the embryoid and the other to the apical part (Figure 5C). Were observed embryoids with the suspensor attached to the laterally side of basal part of embryoids (Figure 5D) and to the central axis of the basal part of embryoids (Figure 4B,C and Figure 5E). It should be noted that the suspensors were not always filamentous, sometimes they were a group of irregularly shaped cells (Figure 5D). The filamentous suspensors were characterized by irregular cell septa and varying cell lengths. This could be observed at all stages of embryoids development (Figure 2G, Figure 3L,M, and Figure 5F).

**Figure 6:**
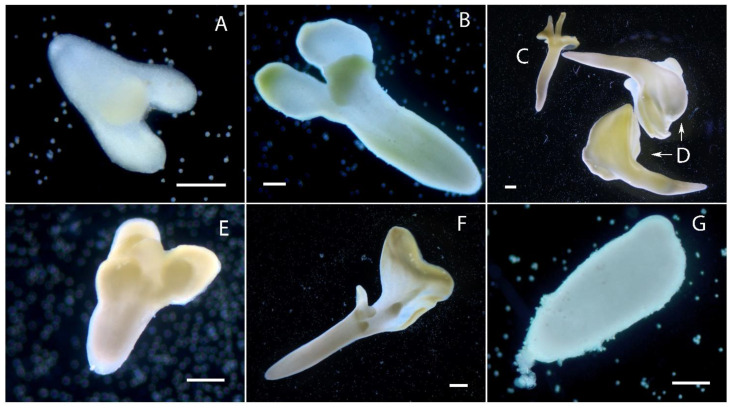
European radish embryoids obtained in microspore culture *in vitro*, with different types of cotyledons development: (**A**–**C**) Embryoids with three cotyledons; (**D**) embryoids with cotyledons with a fused collar shape; (**E**,**F**) embryoids with more than three cotyledons; (**G**) embryoids without cotyledons. Bars = 200 µm.

In the cotyledon stage of embryoid development, aberrant forms of cotyledons were observed. For example, in addition to two cotyledons (Figure 4), embryoids with three (Figure 6A–C) or more apical meristems (Figure 6E,F) or cotyledons with a fused collar shape (Figure 6D) were present in culture, or they were absent altogether (Figure 6G).

**Figure 7:**
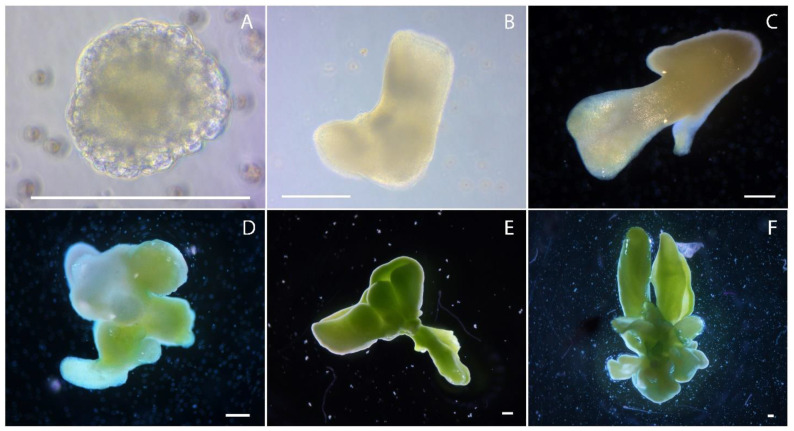
Aberrant development of European radish embryoids obtained in microspore culture *in vitro*: (**A**) Embryoid with abnormalities in the morphology of the outer layer cells; (**B**–**F**) embryoids with different anomalies. Bars =300 µm.

In addition to classical development and partially aberrant development, completely abnormal structures were present in the culture. Figure 7 shows some of them. Abnormality consisted of the absence of polarity (Figure 7A,B,D,E), symmetry in the axis, and orderliness of the apical meristems (Figure 7B,D–F). Abnormalities in the morphology of the outer layer cells were also observed (Figure 7A).

**Figure 8:**
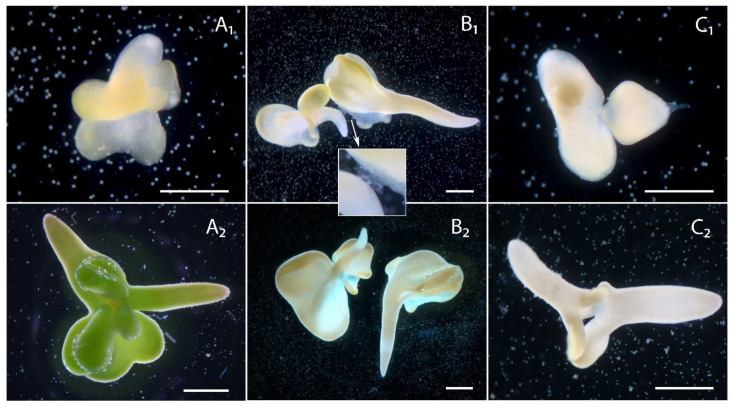
Development of twin embryoids in an *in vitro* culture of European radish microspores: (**A_1_**,**A_2_**) Forms of fused twin embryoids without suspensors on the 13th day of culture and the 23rd day; (**B_1_**,**B_2_**) Twin forms of embryoids joined by thin layer of cells and with suspensor attached to the basal part of one of the embryoids on the 13th day of culture and the 18th day; (**C_1_**,**C_2_**) Forms of fused twin embryoids with a suspensor attached to the basal part of one of the embryoids on the 13th day of culture and the 18th day. Bars = 500 µm.

Twin forms of embryoids were present in the culture. Twins were formed with (Figure 8B_1_,B_2_,C_1_,C_2_) and without (Figure 8A_1_,A_2_) suspensors. The joined of twins looked like a thin layer of cells (Figure 8B_1_) or twins were fused (Figure 8A_1_,A_2_,C_1_,C_2_). Joining and fusion occurred in the apical part of the embryoids (Figure 8A_1_,A_2_,C_1_,C_2_) or in the basal part of one embryoid and the apical part of the other (Figure 8B_1_). During culturing, if the twins were joined by a thin layer of cells and not fused, they subsequently separated from each other by tearing the connective layer of the cells (Figure 8B_2_). Suspensors in twin forms were attached to the basal part of only one of the embryoids (Figure 8B_1_,B_2_,C_1_,C_2_), and we did not observe a pattern in the rate of embryoid development depending on the presence of the suspensor.

**Figure 9:**
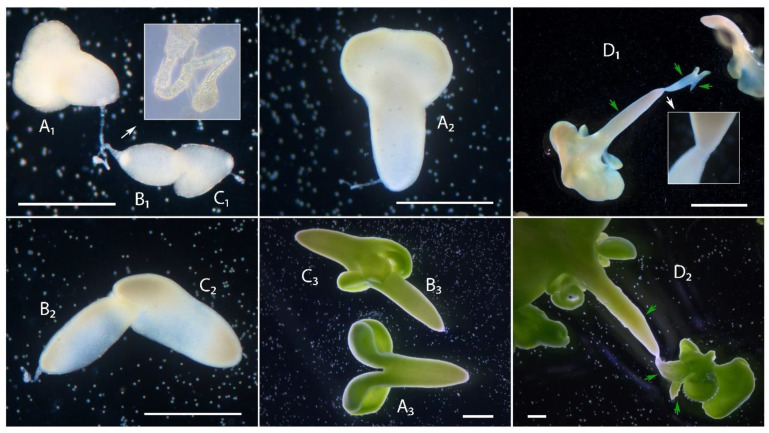
Development of triple twin embryoids in an *in vitro* microspore culture of European radish: (**A_1_**,**B_1_**,**C_1_**) Triple twins, where two embryoids are joined together by the suspensor and the other two are fused on the 14th day of culture, (**A_2_**,**B_2_**,**C_2_**) 18th day, and (**A_3_**,**B_3_**,**C_3_**) 23rd day; (**D_1_**) triple twins are shown where all three embryoids are fused, two basally and two apically on the 18th day of culture and (**D_2_**) 23rd day. The green arrows are the embryoids. Bars = 500 µm.

In addition to twin forms with two embryoids, triple twins were found in culture. In contrast to the twin forms with two embryoids, the triple twins could be joined together by a suspensor. Thus, Figure 9A_1_,B_1_,C_1_ shows triple twins (13th day of culture), where two embryoids are joined together by the suspensor (Figure 9A_1_,B_1_) and the other two are fused (Figure 9B_1_,C_1_). Interestingly, in this case, the suspensors were attached to all three embryoids on the basal side. Then, on the 18th day of culture, as in the case of twin forms joined by a thin layer of cells, we observed a rupture of the suspensor, and the twins separated into one separate embryoid (Figure 9A_2_) and two fused embryoids (Figure 9B_2_,C_2_), which continued to develop fused. Unfortunately, we were unable to identify the early development of the triplet twins, but there was an obvious difference in their developmental stages on day 13 of culture in Figure 9A_1_–C_1_ we can observe cotyledonous, late globular and heart stages of embryoid development simultaneously. However, during growth, the developmental stages of the twins aligned, and on the 23rd day of culture, they were all at approximately the same cotyledon stage of development, ready for transfer to solid nutrient media (Figure 9A_3_–C_3_). The second type of triple twins is shown in Figure 9D_1_,D_2_, in which case all three embryoids were fused, two basally and two apically. Here, we also observed differences in the developmental stages of the embryoids (day 18, Figure 9D_1_), with differences remaining prominent during further development (day 23, Figure 9D_2_). The cotyledons of these triplet twins were abnormal.

**Figure 10:**
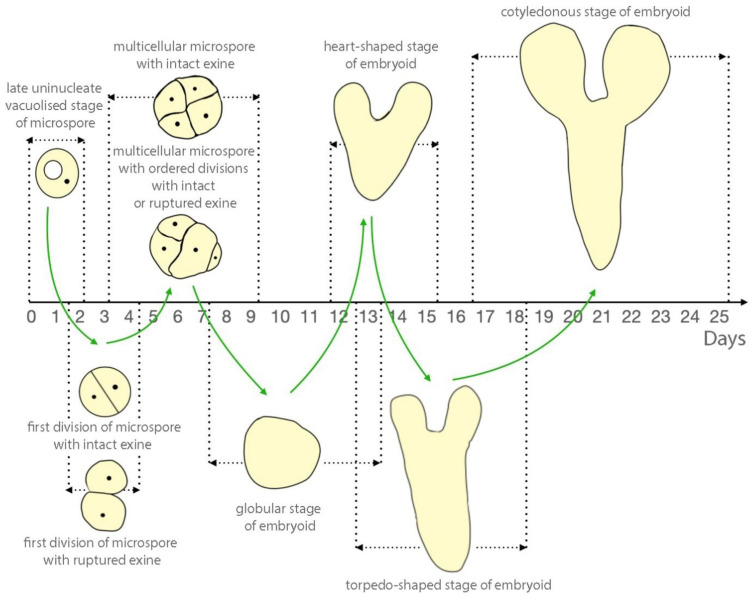
Stages of embryoid development in *in vitro* culture of European radish microspores as a function of culture duration.

As a result of observing the *in vitro* culture of European radish microspores, we drew up a diagram of the dependence of embryoid development stages on the duration of culture, that is shown in Figure 10.

## 3. Discussion

Despite the fact that the process of embryogenesis is actively studied and many aspects of embryogenesis biology for model cultures are already known, many questions remain, and new data continue to appear, which sometimes contradict earlier theories and assumptions.

The structures that emerge during *in vitro* culture of microspores are very diverse, and one can observe a completely different morphology of their divisions depending on the degree and time of the release of microspores from exines, which is considered one of the main regulators of the initial pathways of embryoid development, and the determination of their polarity [25,27]. In our study, we did not sequentially track the formation of each cell structure individually. However, we analyzed the results obtained by comparing the morphology of different structures recorded during the culture of microspores for 30 days, relying on literature data.

We observed different variants of exine ruptures from intact to completely exine-free microspores. It is worth noting that we have not encountered data in the literature on microspores that were already completely free of exines on the second day of culture. However, we believe that the division patterns of such cells should be similar to microspores where there is an extensive exine rupture with impaired cell adhesion, since when the exine is extensively ruptured, it no longer exerts pressure on the microspore protoplast, and therefore the microspore protoplast is in the same condition as when the exine is completely absent [24,28].

Embryogenesis in rapeseed microspore culture has been described in the literature, in which the developmental pathway of microspores with a single exine rupture was studied particularly thoroughly [27]. It was shown that MDE (microspore-derived embryoids) polarity was dependent on exine rupture and was not related to asymmetric cell division, because exine rupture even before the first cell division induced organelle reorganization, which determined MDE polarity and fixed the further apical–basal axis of embryoids. In our studies, this was confirmed. In embryogenic structures of future single embryoids formed from micropores with a single exine rupture, the long division axis (polarity/future apical–basal axis) was always perpendicular to the exine rupture axis. Unfortunately, we have no data to confirm or refute this hypothesis for twin forms of embryoids. However, we can assume that the formation pathways of twin structures attached to each other without a suspensor are not as unambiguous as described by Tang et al. [27], because our studies observed twin embryoids attached to each other, via the apical part of one and the basal part of the other. This suggests that their apical–basal axes were lined up sequentially with each other rather than emerging from a single point, as would be the case if they were formed simultaneously from a microspore that split perpendicular to the rupture of the exine with polarity already fixed and the apical–basal axis of the future embryoids. In the case of such attachment of embryoids to each other, especially considering the fact that they were not at the same stage of development and were connected to each other by a thin layer of cells rather than by a suspensor, we can assume that initially one embryoid was formed and only then did the second, on the surface of the first, which looks like secondary embryogenesis [25].

In our study, it was also confirmed that the embryoid itself can develop both from an exine-coated and exine-free cell, which agrees with Tang et al. [27] and does not fully agree with other data [26,28,29]. In addition, the literature describes that more vacuolized cells developed into a suspensor and more “dense” cells developed into MDE [27,30]. In our studies, we observed a similar scenario of cell development depending on the degree of vacuolization.

Tang et al. [27], investigating rapeseed MDE, suggested that an exine rupture determines the first division axis in most cases due to the pressure difference inside the cell between its regions with and without exine, but in European radish culture, microspore divisions perpendicular to the exine rupture axis were very frequent, suggesting that the first-division axis is hardly dependent on exine rupture.

In contrast to many studies describing classical embryogenesis with an intact exine, where divisions occur haphazardly before its rupture and the apical–basal axis is formed after the exine rupture [22,23,27,30,31,32]. In our observations, some microspores showed ordered divisions in intact exines as early as the first week of culture. 

Microspore divisions with extensive rupture of the exine and those that completely emerged from it resembled several loosely connected cells (loose callus). Callus-like structures were previously thought not to be embryogenic [23,29], but then it was shown that they express several embryo identity genes and are capable of forming histo-differentiated embryoids [24,28]. Corral-Martínez et al. [33] obtained surprising results by tracing the individual developmental pathway of structures with an intact exine, with one exine rupture (compact callus) and complete rupture (loose callus). The loose callus, whose cell morphology was more similar to that of the suspensor cells, was found to be much more likely to form embryoids than the compact callus with a single exine break, and it was the main source of embryoids with rudimentary suspensory cells.

We can confirm that microspores with extensive exine rupture are embryogenic—most structures with suspensor-like cells (days 7–9 of culture) showed no traces of attached exine residues (we described above that we believe that cell divisions of microspores with an extensive exine rupture are similar in their morphology to those of microspores completely free of exine). However, we could not always trace the chronology of the development of all groups of divisions in the initial stages of embryogenesis (before the globular/core stage), so we cannot state whether these structures have developed into complete EDMs. In addition, we have no statistical data to confirm the frequency of EDM formation from specific structures.

In our *in vitro* studies of European radish microspore culture, we observed two developmental pathways of the embryoid with the suspensor. In one, the formation of the suspensor and embryoid occur simultaneously, while in the second, the cells initially divide transversely and form the suspensor, then form the embryoid, which is consistent with data from other researchers [26,27,34]. Interestingly, there are different views in the literature regarding the mechanism of embryoid formation with the suspensor [24,35,36,37,38,39,40]. Supena et al. [26] studied the variants of embryoid formation with suspensors in detail, including twin forms. The researchers performed an interesting experiment where the suspensors were transplanted to a solid medium in which, as the cells of the suspensor were dividing it would bend, and embryoids would form on these bends. In this way, the researchers obtained a chain of embryoids on a single suspensor. These observations suggest that the embryoids were formed due to kinks in the original suspensor and that all cells in the suspensor had the potential to be embryogenic. These studies correlate well with the work of Liu et al. [41], where the embryogenic potential of suspensor cells, which is suppressed by the embryo itself, was confirmed. Supena et al. [26] noted that although they observed various variants of suspensor formation, eventually, after embryoid development and formation of the apical–basal axis, suspensor cells never attached to the apical part of the embryoid. For example, in the case where the embryoid was formed in the center of the suspensor filament, the apical–basal axis was formed perpendicular to the suspensor axis during embryoid formation, so that both suspensor arms remained in the basal part of the embryoid. In such variants, they also observed the formation of twin embryoids with the attachment of the shoulders of the suspensor to their basal parts. From these observations, the researchers concluded the important function of the suspensor in determining the future apical–basal axis of embryoids.

In our study, we observed different locations of suspensor attachment relative to the apical–basal axis of embryoids. We also at the first time encountered attachment of the suspensor to the apical parts of embryoids between the cotyledons and on the side of the apical part. Moreover, we observed embryoids in the central part of the suspensor that formed an apical–basal axis parallel to the axis of the shoulders of the suspensor, so that one suspensor was attached to the basal part and the other to the apical part of the EDM. These observations suggest that the conclusion about the suspensor determines the apical–basal axis of embryoids and is always a marker of the future basal part is not entirely correct.

Furthermore, Supena et al. [26] report that embryoids formed from the central part of the suspensor took several times longer to develop than those formed at one end of the suspensor. This was not confirmed in our observations. There was no pattern in the rate of embryo development, with no difference even when compared to embryoids that developed along the classical pathway without the suspensor from microspores with intact exines, although many researchers in the literature have said that embryoids with a suspensor developed slower than the cotyledon stage of development compared to those without a suspensor [34,41,42,43,44,45,46]. Nevertheless, we observed uneven development of embryoids in general, so at the same culture time we could observe embryoids at different stages. For example, on day 13 of culture, we could observe embryoids at globular, heart-shaped, and torpedo-like stages of development simultaneously. Interestingly, the twin embryoids almost always showed a difference in developmental stages, but their developmental rate was different, because by the cotyledon stage, almost always, their development leveled off. We can also note the faster maturation of European radish embryoids (18–23 days) compared to researchers’ data on the rate of development of rapeseed embryoids (26–40 days) [34]. However, it was noted that up to the globular stage of development, the process of embryogenesis in rapeseed and the European radish proceeds at the same rate, and only then does the rate of maturation of radish embryoids increase in comparison with *B. napus*. We observed the torpedo stage in radishes from day 13 to day 18 of culture, while rapeseed embryoids reach this stage only by day 16–20 [34].

To explain the numerous deviations of European radish embryoids from normal development, additional studies are required. However, there is evidence in the literature that aberrant cotyledons are formed as a result of impaired auxin transport, which can probably also explain our results [35,41,45,46]. The wild ancestors of the radish did not possess a rootstock, which means that edible radish root crop is a young trait that resulted from the selection of mutant forms with impaired cell division [47,48,49]. It is known that phytohormones and their transport are largely responsible for the control of cell division and proliferation. We have already encountered abnormal development of the root system of DH-plants of the European radish obtained in microspore culture *in vitro* [50]. Accordingly, it is quite expected to observe a disturbance of phytohormone transport at the stage of embryoid formation.

This work demonstrates that the embryogenesis of microspores in *in vitro* culture of the European radish is largely similar to that of other Brassicaceae cultures, but there are features that have not been previously described in the literature and require special attention in further studies.

## 4. Materials and Methods

### 4.1. Donor Plants and Growth Conditions

As a material for the study, a variety of the European radish “pink-red with a white tip” (RBK) was taken, as the most responsive variety to embryogenesis [51]. Donor plants were grown in pots filled with a mix of substrate: Peat, vermiculite, and sand (6:1:1), in a growth room under a 16 h photoperiod with illumination at 9000 lux at a constant 19 °C. When the plants were at the 2–3 leaf stage, they started being watered three times a week with 0.1 g/L liquid fertilizer (N-13%, P_2_O_5_-5%, K_2_O-25, MgO-2%, S-8,Fe (DTPA)-0.054%, Zn(EDTA)-0.014%, Cu(EDTA)-0.01%, Mn(EDTA)-0.042%, Mo-0.004%, B-0.02%).

### 4.2. Culture of Microspore In Vitro

We used buds with a size of 3.9–4.2 mm. Surface sterilization of the buds was carried out for 30 s in 70% ethanol, then 15 min in a 50% aqueous solution of the commercial preparation “Belizna” with the addition of Tween-20 (1 drop per 100 mL), followed by three rinses for 10 min in sterile distilled water. Isolation of the microspores was carried out using the protocol for obtaining doubled haploids in *in vitro* microspore culture for the Raphanus sativus [20]. The NLN-13 medium was used as a nutrient medium; before being introduced into the culture of microspores, the buds were kept for 24 h in a refrigerator at 4; after the isolation of micropores, the Petri dishes were kept for 48 h at a temperature of 32 °C, then transferred to a thermostat with a temperature of 25 °C.

The final pellet was suspended in NLN-13, and the microspore density was adjusted to 12,000 cells per ml. The microspore suspension was then poured into Petri dishes (5 mL per 60 mm Petri dish).

### 4.3. Data Visualization

To visualize the living culture, an inverted microscope Primo Vert, Zeiss, binocular Stemi 508, Zeiss, and camera Axiocam 305 color, Zeiss were used. Petri dishes with live microspore culture were examined every 2–3 days, starting from the first day of culture, and interesting data were photographed.
